# Supplementary material for: Serological profiles of pan-coronavirus-specific responses in COVID-19 patients using a multiplexed electro-chemiluminescence-based testing platform
Source: PLoS One. 2021 Jun 3;16(6):e0252628. doi: 10.1371/journal.pone.0252628 (PMC8174743; doi:10.1371/journal.pone.0252628)
Supplement: S1 Fig — (PDF) [file pone.0252628.s001.pdf]

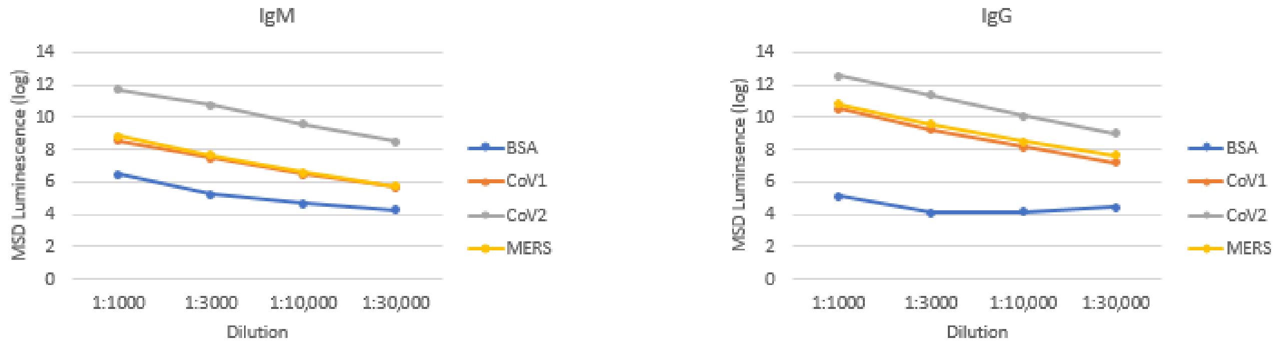

S1 Fig: Sensitivity and specificity of assay to detect SARS-CoV-2-specific antibodies. Serial dilutions (indicated on x-axis) of COVID19 patient samples (n=10) were testing for CoV-specific IgM (left panel) and IgG (right panel) in the 10-plex assay; plate antigens: BSA (negative control), spike protein of SARS CoV1, SARS-CoV-2, MERS CoV. Data expressed as mean luminescence signal (log).
