# Supplementary material for: Serological profiles of pan-coronavirus-specific responses in COVID-19 patients using a multiplexed electro-chemiluminescence-based testing platform
Source: PLoS One. 2021 Jun 3;16(6):e0252628. doi: 10.1371/journal.pone.0252628 (PMC8174743; doi:10.1371/journal.pone.0252628)
Supplement: S2 Fig — (PDF) [file pone.0252628.s002.pdf]

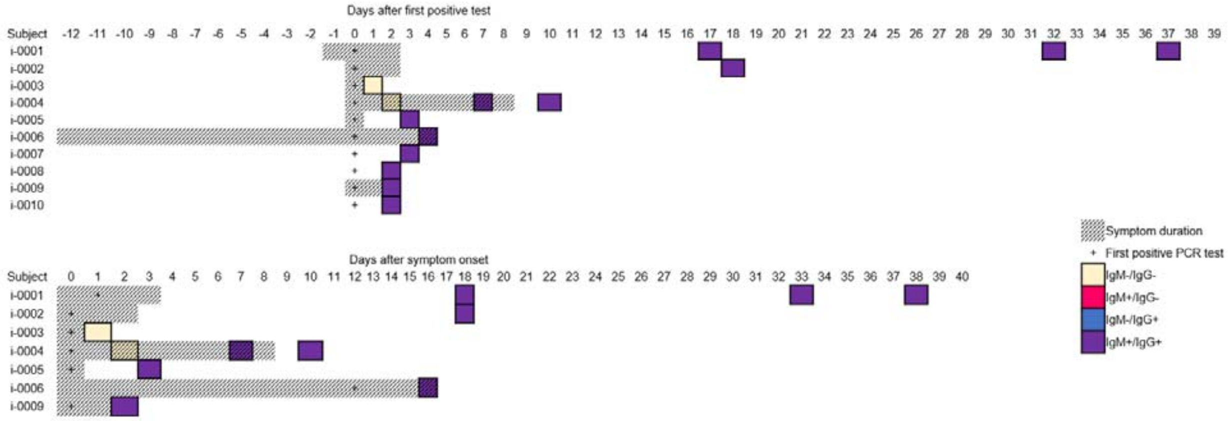

S2 Fig: IgM and IgG seropositivity with respect to disease progression. Seropositivity with respect to disease progression is shown for time after first postive PCR test (top) and time after sympton onset (bottom), with time of first positive test (‘+’), symptom duration (shaded), and seropositivity results (seronegative, tan; IgM+/IgG-, magenta; IgM-/IgG+, blue; IgM+/IgG+, purple).
