# Supplementary material for: Serological profiles of pan-coronavirus-specific responses in COVID-19 patients using a multiplexed electro-chemiluminescence-based testing platform
Source: PLoS One. 2021 Jun 3;16(6):e0252628. doi: 10.1371/journal.pone.0252628 (PMC8174743; doi:10.1371/journal.pone.0252628)
Supplement: S1 Table — (PDF) [file pone.0252628.s003.pdf]

S1 Table: Age and sex of Control Subjects

Subject ID	Age Range (y.o.) [Sex]
PG3-0061	60-69 [M]
PG3-0062	30-39 [M]
PG3-0064	40-49 [F]
PG3-0066	50-59 [M]
PG3-0067	30-39 [M]
PG3-0068	40-49 [M]
PG3-0069	30-39 [F]
PG3-0070	40-49 [M]
