# Supplementary material for: Serological profiles of pan-coronavirus-specific responses in COVID-19 patients using a multiplexed electro-chemiluminescence-based testing platform
Source: PLoS One. 2021 Jun 3;16(6):e0252628. doi: 10.1371/journal.pone.0252628 (PMC8174743; doi:10.1371/journal.pone.0252628)
Supplement: Attachment — Submitted filename: Response to Reviewers.pdf [file pone.0252628.s005.pdf]

Silver Spring, 10 Feb 2021

Dear Dr. Roques,

We thank you and the reviewers for the thorough evaluation of our manuscript. Right up front, we would like to address the main concern of the reviewers that we are comparing very different populations. This is a misunderstanding of the cohorts as both, the control and COVID-19 subjects, were recruited from the same site and same study population (U.S. Department of Defense military, civilian, and contractor population working at Camp Humphreys/South Korea). We updated the Methods to ensure that this is clear. Please find below the point-by-point responses to the reviewers' and the editorial team's comments.

We hope that the revisions and the clarifications make our manuscript suitable for publication in PLOS ONE.

Detailed responses to the editorial team:

- **Funding statement:**

The work was funded by the Military Infectious Disease Research program (MIDRP), which was not in the online database of funders. The funders did not have any influence on this study and the experimental plan.

- **Revision of figures**

We have reformatted the figures using the PACE website and uploaded the revised files.

- **Details of experimental work**

We have updated our protocol to include catalog numbers and other details as outlined below. It should be noted that we have followed the manufacturer's (Mesoscale) instructions for the qualified assay. We now describe the computational analysis in the manuscript. If the reviewers/editor feel that the readers would benefit from either portion of the experimental work or the computational analysis being further detailed in a separate protocol, then we will deposit a description in protocols.io.

- **Journal requirements:**

(1) Please make sure that your manuscript meets PLOS ONE's style requirements.

We have made the requested corrections.

(2) Please provide a sample size and power calculation in the Methods, or discuss the reasons for not performing one before study initiation.

We have added the following statement to the Materials Section (lines 129-133): "*Samples from 10 COVID-19 subjects and 8 Control subjects, matched by study location and population, were obtained and compared to a similar number of pre-pandemic samples (10) using the multiplex ECLIA assay. As this was a retrospective analysis of COVID-19 samples collected during a public health investigation of a local outbreak, no a priori power calculation was carried out.*"

(3) ***Supplementary materials are referenced in your manuscript but appear to be missing. Please upload these as supplementary files.***
We apologize for the omission – an error during the initial submission process. We have uploaded the missing figure S1 Fig, added new supplementary materials; (1) a supplementary S1 Table (demographics of the population control as well as pre-COVID-19 samples), and (2) S2 Fig (IgM and IgG seropositivity with respect to disease progression); (3) spreadsheet with the raw serological data for readers to download and reproduce our results.

(4) ***To comply with PLOS ONE submission guidelines, in your Method section, please provide***
   a) ***the source, catalog numbers, and the dilution of the SULFO-TAG antibodies in your study***
   line 169 – we have added the details on the antibodies: "…SULFO-TAG either with anti-human IgG (Cat.No D20JL, MSD) antibody or anti-human IgM (Cat.No D20JP, MSD) was diluted to 2 µg/ml in Diluent 100…"
   b) ***the catalog/identifying numbers for the two commercially available pooled plasma samples:***
   line 143 – we have added the order information (GeminiBio (GemCell™ U.S. Origin Human Serum AB, Cat.No 100-512). We obtained the items to test them for their ability to support tissue cultures and received several lots to test in 2018. These two samples were aliquots of this lots.
   c) ***Sequence or accession numbers of the antigens used in your study.***
   We have added the requested information (lines 154-159).

5) Funding information

As mentioned above, the work was funded by MIDRP and we have deleted the section in the acknowledgements. We would appreciate if we could revise this in the online submission system.

6) Data access

We have revised the statement to:" *The serological data (expressed as Luminescence signal) are provided in S2 Table. These data are raw data and were log-transformed for the downstream analysis described in the manuscript"* Lines 478-479

7) Ethics statement

We have removed the section and added the information to the Materials section (lines 145-149).

8) Please include captions for your Supporting Information files at the end of your manuscript, and update any in-text citations to match accordingly.

We have made the requested revisions.

Discrepancy in titles between manuscript and submission system

We have adjusted the title in the submission system to reflect the title in the manuscript file.

REVIEWER 1 COMMENTS:

Major Points:

1) Population study and selection criteria, and sampling: The covid-19 and non-COVID-19 patients are enlisted at the same site and in the pandemic time period. However, the sampling process needs to be precise. The selection criteria has to be the same between these both populations in order to perform comparative study.

Control and COVID-19 subjects were recruited from the same site and study population (U.S. Department of Defense military, civilian, and contractor population working at Camp Humphreys). We updated the Methods to reflect this:

> "COVID-19 and Control subjects were drawn from the same overall population: the U.S. Department of Defense military, civilian, and contractor population working at Camp Humphreys." Lines 124 ff

We also added a paragraph in the Discussion section highlighting the limitations to the present study (lines 446 ff):

> "*There were several limitations to the present study. First, the sample size is relatively small and as such the study is intended primarily to demonstrate feasibility of the multiplex ECLIA assay. Second, the samples were obtained through a public health investigation of a local outbreak in Camp Humphreys, and, thus, largely consists of 'convenience' samples. While we matched Control subjects to the same location and study population, a rigorous case-control study was infeasible in the midst of an emergency outbreak response. Still, the samples reflect diversity in disease onset and severity that parallel samples collected in real-world serosurveillance efforts. Third, with some exceptions, the study did not include longitudinal sample collection, which limits its findings with respect to disease progression. Fourth, while COVID-19 and Control groups were matched by site and population, pre-pandemic samples were obtained from a sample collection protocol carried out domestically, in Maryland, and ,thus, provides an imperfect pre-pandemic comparison to the pandemic samples.*"

2) The negative status of non-Covid-19 patient (working in health center) seems only based on the observation (clincal forms) (evaluation bias) and/or the paitent declaration during the interview (no risk of exposition?) (= memory bias). While the prevalence of COVID-19 in Health Care Worker is low, providing evidence of (RT-qPCR) tests for non-covid-19 group is recommended.

Control samples were tested for COVID-19 using the same diagnostic assay as the COVID-19 patients. We regret this omission, and it is now included in the Methods (Line 134-135):

> "*All Control subjects were also tested via nasopharyngeal swab and RT-PCR and confirmed to be negative for COVID-19 at the time of sample collection.*"

3) The military (covid-19 group) as well as the health care persons (non covid-19 group) are often considering as healthy and volunteer persons in participating in study compared to

the general population. This behavior may impact on serological results and needs to be taking into consideration (analyses and discussion).

We have included age range and sex for COVID-19 and Control subjects in Table 1 and in S1 Table. Subjects in this study were *not* exclusively military, and included U.S. Department of Defense civilians and contractors thus reflecting a wide demographic range.

4) The selection of pre-pandemic samples from several sources in the United States in 2019 is also questioned (Random or voluntary strategies?). In addition, epidemic information about coronavirus circulation during this 2019 summer would allow to determine whether the exposure to others CoV species differs between pre-pandemic samples and pandemic samples (Covid-19 (+) and Non-Covid-19 (-)) and enrich the cross-reactivity analyses.

Selection of pre-pandemic samples was based purely on availability of samples obtained from a prior WRAIR blood collection protocol, and was thus effectively random, within the geographic and demographic constraints of the local population. We updated the method to reflect that:

> "*Pre-pandemic samples were obtained from a WRAIR blood collection protocol (WRAIR#2567), based on sample availability, from August 2019 conducted in Silver Spring, Maryland.*" Lines 140 ff

Since the COVID-19 and Control samples were collected from a Department of Defense military and civilian population that is relatively highly mobile, it would not be possible to determine general trends in pre-pandemic coronavirus exposure in these individuals with diverse geographic history.

5) The lack of homogeneity in the inclusion criteria between 3 groups making difficult the value of antibody responses and their interpretation. Sex and ages: as influencing the antibodies response (i.e.=kinetic, duration, level), the sex and ages need to be noticed in the group.

Sex and age range in COVID-19 group and Control group now included in Table 1 and S1 Table respectively.

6) In addition to table 1, the illustration of patient features through a timeline-scale would better link the diseases progression, onset with the antibody responses and potentially other determinants factors.

We have now added a timeline for the COVID-19 subjects that includes symptomology, testing, and seropositivity as determined by the ECLIA assay in S2 Fig. We added the following paragraph to the Results section (lines 342 ff):

> "*Analysis of IgM and IgG seropositivity in COVID-19 subjects, with respect to time from first positive test and onset of symptoms (S2 Fig) showed that (1) seronegative results were only found in two cases (i-0003 and i-0004) where the sample was collected within two days of the onset of symptoms and that (2) all samples that were seropositive by IgM were also seropositive by IgG, as measured by response to the SARS-CoV-2 spike protein. This apparent simultaneous seroconversion was seen as early as two and three days after onset of symptoms (i-0009 and i-0005, respectively).*"

Minor points:

- **Severity level determination: The illness definition and the levels severity remain uncleared for the covid-19 group: is it related to a score scale and performed by a same medical person (table 1: page 186)?**

We provided additional detail in the Methods section to address this question:

> "*COVID-19 disease severity was assessed as asymptomatic, mild (symptomatic, but not interfering with daily activity), moderate (interfering with daily activity, but not requiring hospitalization), and severe (preventing daily activity and requiring hospitalization).*"

- **The 334-347 discussion paraph, including particular objectives, should be moved in the introduction than in the conclusion to facilitate the results analyses and authors goals.**

We removed that paragraph as we felt it was redundant with material in the Introduction

- **Results presentation in only 2 major parts "IgM/IgG characterization" and "the cross reactivity events" might facilitate and open the discussion and subchapters could be suggested.**

We opted to keep the Results section organization as is since sub-sectioning the results would fragment the data presentation too much.

- **The authors claim that by using a larger data set, ELCIA would have high potential for predicting acute infection status and exposure of an individual from their serological data. If is it true: how to explain the difference between id-003 and id-009?**

The assay was able to detect seroconversion in 3 of 5 cases where samples were collected within seven days of symptom onset (S2 Fig, bottom panel), showing that detecting acute infection using this assay is possible, even in mild cases. The observation that not all samples collected in this time span showed seroconversion highlights the limitations of using a serology assay to detect early infection.

- **As arguing the authors, the absence of antibodies response to Subject i-0003 may be related to mild clinical presentation mild symptoms and/or the delay of serconversion (only one day following initial test positivity). If so, what about the interpretation of the id-009 results, showing similar clinical profile as the id-0003 patient (215-218)?**

It is common for different individuals to have different time courses with respect to disease progression and seroconversion. Therefore, it is not unusual for one subject to seroconvert one day before another subject with a similar clinical profile.

- **As mentioned above, the authors suggests that the delay in antibodies response is related to the low clinical symptom: How explaining the IgM and IgG profiles of the id-007, id-008, id-0010? = asymptomatic patients whose samples, collected + 2 days after PCR (+), are associated to significant antibodies response?**

It is difficult to assess disease progression in asymptomatic subjects because they do not have a time of onset of symptoms from which to compare with other subjects. Time of first positive test in asymptomatic subjects is, to some extent, arbitrary, based on when external circumstances (routine testing, contract tracing, etc.) prompted them to get tested.

- **To argue in the IgM/IgG conjugated detection importance in Covid-19 management and/or survey, discussion should be enriched with additional related references.**

We believe that integrating multiple serological measurements in serosurveillance and diagnostic efforts is quite novel, very powerful, and not yet established in the literature, and we hope that this study will spur further research efforts in this direction.

- **The authors address possible limitations of the research, including cross-reactivity To gain insights into the pre-existing immunity Covid induced antibody responses impact, the distinct patterns of cross-reactivity in IgM and IgG responses are plenty explored in the literature, such as the multiplex tools use, as here.**

We could not find other examples of published studies exploring cross-reactivity of IgM and IgG responses in COVID-19, except those already cited in the Discussion section.

- **The cross reactivity appears higher with IgG SARS-CoV-2 than the IgM SARS-CoV-2 binding antibodies. The author provided a long analysis in the cross reactivity trouble between coronavirus species. SARS-CoV-2 IgG antibodies may be cross-reactive with SARS-CoV-1 and MERS-CoV: 1) 33% of CoV-2-seropositive COVID-19 samples were also seropositive for MERS-CoV, 2) 50% IgM seropositivity for MERS-CoV was detected the Control Group (275-277). In view of the % in MERS/SARS COV-2 cross reactivity, the MERS serological status of control population should be clarify : Is it a lack in specificity of the ECLIA approach or a real high MERS prevalence in control populations?**

We stated in the Discussion that we believed it was the lack of specificity in the ECLIA and that validation with each CoV antigen was necessary to establish threshold values for seropositivity (lines 384 ff):

> "*Here we provide this validation for SARS-CoV-2 antigens using samples known to be exposed to SARS-CoV-2 and utilized a single threshold for defining seropositivity based on negative controls, but cross-reactivity in antibody responses between the CoV antigens necessitates individual validation of responses to each antigen to maximize specificity. This limitation is highlighted in the apparent 50% IgM seropositivity for MERS-CoV in the Control Group. While there was a MERS outbreak in South Korea in 2015, there were only 186 confirmed cases in that outbreak [22] and a more likely explanation is that this reflects a cross-reactivity from immunity to a related beta coronavirus.*"

- **Confunding factors: As mentioning in the results revision, details about patients (sex and age) may help authors as well as reviewers by arguing some exceptions instead of criticizing the tools accuracy.**

We analyzed for significant differences in age-range and sex and did not find any. A study of this size is not sufficiently powered to identify significant differences in serological data with respect to sex and age.

- **The cross relationship between antibodies profile and clinical presentation needs to be discussed according to some host determinants. The sampling design impacts also in the way.**
  -Example concerning sex determinant: the proportion of female is lower in the military population compared to general population
  -Example concerning military group: this population is better physical fitness, influencing the host responses to pathogen.
  -Example concerning control group: even not being directly in contact to Covid (+) patients, people working in hospital have usually better style of life than the general population (or take better care of their health).
  All of these justify the above revision request by giving details about the study of population and arguing on the criteria selection etc...

The COVID-19 and Control subject population in this study are not exclusively military and includes civilians and contractors and age and sex are now provided in Table 1 and S1 Table. A systematic assessment of differences in serology between military and civilian populations is outside of the scope of this effort. Regarding the concern related to the lifestyle of the study population not being representative of that of the general population it should be noted that they represent individuals working at a military base in various functions, and are not necessarily healthcare workers).

- **By combing IgG and IgM results, the authors success in mapping the course of this subject's antibody response from the pre-COVID-19 or non-infected region to the COVID-19 region independently to clinical symptoms. This view may help in vaccine implementation as well as vaccine efficacy. Nevertheless, this is not fully achieved in view of the id-003 antibody response (low antibody response, low duration or delay?). The results of serological profile of id-0003 need to be checked with others tools (ELISA commercial kit or ELISA in house) to conclude on the ELCIA tool performance/limitation.**

The reviewer points out an important aspect of such analyses: It is not uncommon to find COVID-19 subjects that have not seroconverted, particularly early in the disease course. The sample for subject i-0003 was collected one day after symptom onset. The ECLIA assay is much more sensitive than a regular ELISA (we had previously published formal, extensive comparisons of the platforms) and in the absence of an ECLIA signal, it is highly unlikely that any ELISA would be able to detect a signal.

- **The id-003 data calls to future research on IgA profile and/or seroneutalization. It should be important to mention that the diseases progression depends on the seroneutralization antibodies pool as well as the IgA kinetics (i.e. IgA secretion in local). Unfortunately, the authors don't open up enough perspectives.**

We agree with the reviewer that these functional readouts will provide additional, important insights, but would like to point out that binding-based antibody assays are common in both

diagnostic and serosurveillance studies. The relationship between binding and neutralization is a substantial undertaking that is well outside the scope of the present study. It would be appropriate for a follow-up study that will require a different sample collection protocol.

- **The authors could bring more explanations in why: 1) this new multiplex coronavirus antigen evaluation may change the covid-19 survey; 2) the electro-chemiluminescence assay platform (serological high-throughput testing of sera/plasma) may support the current molecular covid-19 diagnosis?**

We describe in the Discussion that a multiplex assay has the potential for greater specificity in serosurveillance over standard single-antigen approaches and also enables for characterization of immunity across multiple coronaviruses to determine if and how such immunity might contribute to COVID-19. In addition, the approach we describe enables significant sample sparing and a high level of reproducibility.

REVIEWER 2 COMMENTS:

(No major comments, minor comments addressed in manuscript text)
